# Poly(ionic liquid)/Wood Composite-Derived B/N-Codoped Porous Carbons Possessing Peroxidase-like Catalytic Activity

**DOI:** 10.1021/acsomega.4c06102

**Published:** 2024-09-03

**Authors:** Sadaf Saeedi Garakani, Kanglei Pang, Elnaz Tahavori, Anuja Pradip Nawadkar, Özlem Uguz Neli, Jiayin Yuan

**Affiliations:** Department of Materials & Environmental Chemistry, Stockholm University, Stockholm 10691, Sweden

## Abstract

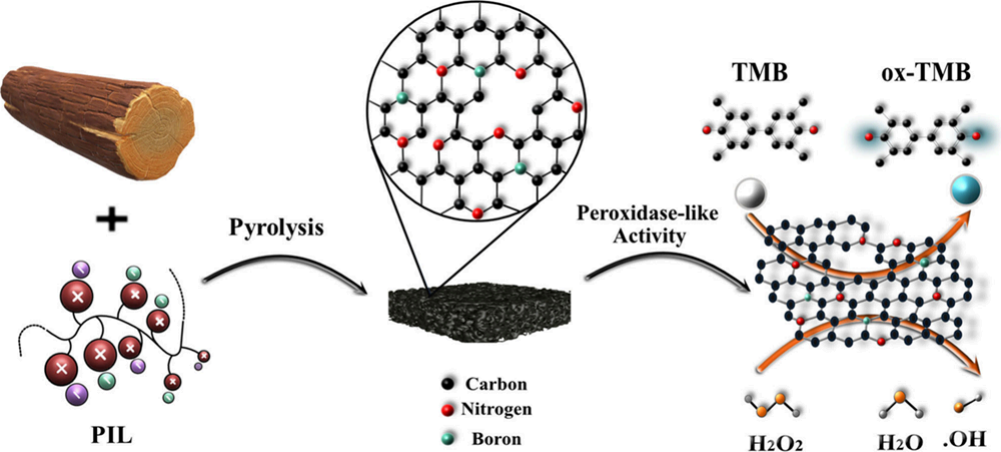

The pursuit of efficient and cost-effective metal-free heterogeneous catalytic systems remains a challenging task in materials research. Heteroatom-doped carbonaceous materials are increasingly recognized as powerful metal-free catalysts, often demonstrating catalytic performance comparable to or even surpassing metal-based alternatives. This is attributed to their tunable physicochemical properties, tailorable structural features, and environmentally friendly profile. In a straightforward single-step synthetic approach, we utilized wood as an eco-friendly and renewable carbon source, in conjunction with a poly(ionic liquid) as a heteroatom source and pore-making agent. The combination of both biobased and synthetic polymers in this method yielded sustainable, high-performance catalysts characterized by enhanced stability and reusability. The inclusion of sacrificial pore-inducing templates resulted in the formation of abundant defects serving as catalytically active sites, while codoping with boron and nitrogen further enhanced these sites, significantly impacting catalytic activities, as established by peroxidase-like activity in this study. The optimized codoped porous carbon membrane exhibited excellent peroxidase-type activity and catalyzed the oxidation reaction of 3,3′,5,5′-tetramethylbenzidine by hydrogen peroxide. This high activity was largely due to the dual heteroatom codoping effect and the mixed micro/macroporous structure of the membrane. Our work presents a versatile and eco-friendly method for fabricating hierarchically porous B/N codoped carbon membranes, offering a manageable, convenient, and recyclable biomimetic artificial enzyme with superior catalytic capabilities. This work introduces a practical and robust colorimetric method that can be used in healthcare and environmental rehabilitation.

## 1 Introduction

Peroxidase enzymes, renowned for their high specificity, sensitivity, and reliability, are frequently utilized in colorimetric sensors. Nevertheless, challenges persist in achieving stability, low production costs, and enabling scalability for large-scale applications. Carbonaceous materials have become favorable substitutes for peroxidase enzymes, given their cost-effectiveness, abundant availability, facile synthesis, tunable properties, and chemical stability.^1^ They have the potential to imitate the activities of peroxidases and assist the oxidative processes such as the oxidation of 3,3′,5,5′-tetramethylbenzidine (TMB) by hydrogen peroxide (H_2_O_2_). Despite the great potential, the carbon-based enzymes are equipped with low-to-modest catalytic activity, retarding their use in a wide scope. To tackle such an issue, there has been extensive exploration of doping carbon materials by heteroatoms to raise their catalytic power.^2^ The merger of doping of heteroatoms with dense hierarchical pores has promised to enhance the activity of carbonaceous enzyme-like catalysts.

Recently, substantial attention has been directed to metal-free heteroatom-doped carbons owing to their unique electronic properties. The attributes of heteroatoms, such as atomic size, electronegativity, and charge density, play a crucial role in shaping their bonds with carbons and, consequently in modulating their corresponding physicochemical properties.^3^ For instance, nitrogen (N) is commonly used to dope carbon due to its prevalence and compatible atomic size to carbon, enabling the formation of C–N covalent bonds. N as a dopant enhances specific functions of carbon materials, e.g., conductivity, oxidation resistance, and catalytic activity.^4^ Consequently, N-doped carbon materials plus the related composites or hybrids have been widely documented for their enzyme-mimicking catalytic activity. For example, Lu et al. have fabricated Fe_3_O_4_/nitrogen-doped carbon composite nanofibers and studied their application as an efficient platform for detecting H_2_O_2_ and ascorbic acid.^5^ Zhu’s team has reported the preparation of porous Pt/N-doped carbons in a honeycomb-like morphology with excellent peroxidase-like catalytic activity.^6^ Furthermore, boron (B) atoms in a doping state in the carbon network can typically adopt an in-plane doping model, i.e. in a stable planar configuration that maintains the *sp*^2^ hybridization as BC_3_. Despite a longer bond in C–B than C–C in the *sp*^2^ hybridization, strong polarization mitigates mechanical stress. Each heteroatom uniquely modifies carbon materials in a different manner.^7^ Harnessing the potential of B as a carbon dopant is challenging due to its oxyphilic nature to be readily oxidized in contact with O_2_ into boron oxide.^8^ Heteroatom codoping, especially in electrocatalysis, supercapacitors, photoelectrochemistry, and sensing, is significant for synergistically tailoring catalytic properties, ensuring desirable activity, stability, and selectivity. Liu et al. prepared a cost-effective B/N codoped mesoporous carbon (BNMC), which was efficient in electrochemical CO_2_ reduction with high Faradaic efficiency and low overpotential. They proved that the codoping effect and the mesoporous structure contributed jointly to its excellent catalytic activity.^9^

The physicochemical properties of heteroatom-doped carbonaceous materials and their chemical compositions are strongly influenced by the chemical nature and microstructure of precursors. Nature, through millions of years, has developed efficient strategies to create well-structured materials, exemplified by e.g., wood’s cellular structure. This structure, with interconnected pores and orientation, in addition to its renewability and low cost, makes wood a favorable carbon precursor for applications requiring high surface area and low diffusion resistance.^10,11^ To expand the potential of wood as a carbon source, additives can be added to modify the physical and chemical properties of wood-derived carbons. For instance, with the abundant heteroatom content, poly(ionic liquid)s (PILs), formed through the polymerization of ionic liquid monomers, can act as a N and B source for introducing targeted dopants into porous carbons.^12^ Moreover, PIL facilitates the creation of additional pores into the carbon matrix via a catalytic degradation process of biomaterials.^13^

Herein, we established a straightforward wood-based approach to produce B/N codoped porous carbon membranes (referred to as “B/N–C″) via sequential pyrolytic treatments. The resulting B/N–C catalysts exhibited remarkable peroxidase-like catalytic activity when applying H_2_O_2_ to oxidize TMB, attributed to the effects of heteroatom doping, high conductivity, and the porous structure. This work indicates the remarkable potential of B/N–C as peroxidase catalysts, which present great prospects for biomedicine and biosensors.

## 2 Experimental Section

### 2.1 Materials

Balsa wood was received from Material AB, Sweden. 1-Vinylimidazole (99%), and tetrahydroxydiboron were purchased from Alfa Aesar. KPF_6_ was obtained from Acros Organics. Bromoacetonitrile (95%) was acquired from TCI Europe. Lithium bis(trifluoromethane sulfonyl)imide (LiTFSI, 99.95%) was received from Io-li-tec. NaClO_2_, NaOAc, 3,3′,5,5′-tetramethyl-benzidine (TMB), l-ascorbic acid, and FeCl_3_ were received from Sigma-Aldrich. N,N-dimethylformamide (DMF) was purchased from Honeywell. H_2_O_2_ was acquired from VWR International. All chemicals were used without any further purification. Solvents were all of analytical grade.

### 2.2 Poly(ionic liquid) (PIL) Synthesis

The precursor, poly(1-cyanomethyl-3-vinylimidazolium bromide) (PCMVImBr), with Br^–^ as the counteranion, was synthesized following our previously published procedure.^14^ To verify the chemical structure, proton nuclear magnetic resonance (^1^H NMR) spectroscopy was used to analyze the poly(ionic liquid), and the ^1^H NMR spectrum in Figure S1 matches well with its chemical structure. Subsequently, poly(1-cyanomethyl-3-vinylimidazolium bis(trifluoromethane sulfonyl)imide) (PCMVImTFSI), a PIL with a larger sized anion TFSI^–^ as counteranion, was obtained through a salt metathesis reaction of PCMVImBr with LiTFSI in an aqueous solution. The salt metathesis reaction involved the dropwise addition of a LiTFSI aqueous solution into a 1 wt % PCMVImBr aqueous solution. The Br/TFSI molar ratio in the final mixture was set as 1/1.15. The solid product was separated, and rinsed with water. The product was dried to constant weight at 70 °C under vacuum.

### 2.3 Delignification Reaction of Wood

The used Balsa wood has a density of 123 kg m^–3^. Prior to delignification, it was sliced into thin membranes of controlled thickness by a cutter (secotom-50). The cutting was conducted in a way to align its direction perpendicular to that of the growth of trunk. Before the reaction, the wood slices were annealed at 80 °C for 10 hs. To remove hemicellulose and lignin in part, sodium chlorite (1 wt %) in an aqueous acetate buffer solution (pH 4.6) was used to treat the wood slices for 6 hs at 80 °C. Following the reaction, the samples were washed first with pure water and then ethanol. The samples were finally placed under ambient conditions and dried until constant weight.

### 2.4 Synthesis of the Carbonaceous Catalyst B/N–C

In a representative test, 0.850 g of the poly(ionic liquid) PCMVImTFSI and 46.8 mg of tetrahydroxydiboron were mixed and dissolved in DMF (8.5 mL), where the imidazolium/hydroxyl molar ratio was set as 1/1. A wood membrane after delignification (425 mg in mass) was drop-coated by the above mixture solution and then dried at 80 °C for 2 hs. The resulting membrane was placed in an aqueous NH_3_ solution (0.25 wt %) for 2 hs to form a porous layer of the cross-linked polymer on the surface of the porous wood. Afterward, the membrane was rinsed with deionized water thrice, and dried to constant weight at room temperature. Subsequently, the modified membrane was heated at a heating rate of 3 °C min^–1^ to 900 °C under vacuum, and maintained at this temperature for 1 h. Finally, it was cooled down in 12 h to ambient temperature.

### 2.5 Assessment of Peroxide Catalytic Activity

Peroxide catalytic activity was assessed by mixing 40 μL of the suspension of the as-made B/N–C at a concentration of 3 mg mL^–1^ with 40 μL of a TMB solution at a concentration of 15 mM at room temperature in DMSO. The mixture solution was injected into 3 mL of an acetate buffer solution (pH = 4) that contained 60 μL of H_2_O_2_ (30 wt %). The oxidation reaction of TMB by H_2_O_2_ using the carbonaceous catalyst was monitored at λ = 652 nm in a 10 min reaction. Control samples, i.e., TMB + H_2_O_2_ (without carbonaceous catalyst) and TMB + carbonaceous catalyst (without H_2_O_2_) at the same concentration, were included for comparison. Along the reaction, the solution was measured by a UV–vis–NIR spectrophotometer (Agilent Technologies). The pH tolerance of the catalyst was examined in a wide pH range of 2.0–9.0 at ambient temperature under predefined concentrations. In a similar manner, the temperature tolerance of the catalyst was investigated at varied temperatures in the range of 20–50 °C at pH = 4.

### 2.6 Analysis of Reaction Kinetics

To study the kinetics of reactions, the absorbance at λ = 652 nm was recorded at an interval of 3 min in a scanning mode. Steady-state kinetics were monitored by applying TMB and H_2_O_2_ as substrates. For the calculation of kinetic parameters, we changed the TMB concentration but maintained the H_2_O_2_ concentration the same for the tests, and *vice versa*. To analyze the kinetic data, we employed the Michaelis–Menten equation, as shown in eq 1.
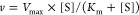
1TMB’s molar attenuation coefficient at 652 nm was determined as 39,000 M^–1^ cm^–1^. In eq 1, *v*, *V*_max_, [S], and K_m_ stand for the initial reaction velocity, the maximum reaction velocity, the substrate concentration, and the Michaelis constant, respectively. All experiments were conducted in colorimetric dishes of 1 cm in thickness.

### 2.7 Analysis and Characterization

The phase structure of the carbonaceous catalysts was studied on an X-ray diffractometer with Cu K_α_ radiation (λ = 1.5418 Å, PANalytical X’Pert Pro) in the range of 5° - 90° which was scanned at a rate of 0.2°/min. Proton nuclear magnetic resonance (NMR) spectra were collected on a Bruker DPX-400 spectrometer operating at 400 MHz at room temperature, using DMSO-*d*_6_ as solvent. N_2_ adsorption/desorption isotherms were operated at 77 K on the micromeritics ASAP 2020 (Accelerated Surface Area and Porosimetry system). Prior to the tests, samples were heated to and maintained at 373 K under vacuum for 7 h for degassing. To access the surface area, we applied the Brunauer–Emmett–Teller (BET) equation. Raman spectroscopy was recorded on a Horiba Labram HR system on a laser at an excitation wavelength of 532 nm. The microscopic structures of catalysts were analyzed on a scanning electron microscope (SEM, JEOL 7000F) which was conducted with an accelerating voltage of 10 kV. The SEM specimens were sputtered by a ultrathin layer of gold prior to imaging. Transmission electron microscopy (TEM) images were collected on a JEOL JEM-2100 microscope which was conducted at an accelerating voltage of 200 kV. To study the constituent elements, energy-dispersive X-ray (EDX) spectrometer equipped on the TEM equipped was applied for the elemental mapping. Characterization of chemical bonds was carried out by ESCALAB 250Xi X-ray photoelectron spectroscopy (XPS). The catalytic processes were monitored on UV–vis–NIR spectrophotometer (Agilent Technologies).

## 3 Results and Discussion

Boron and nitrogen codoped porous carbons in a membrane shape were termed B/N–Cs here. They were synthesized via straightforward carbonization of delignified Balsa wood as an environmentally friendly carbon source. Prior to pyrolysis, the delignified Balsa wood was precoated by a mixture solution of a poly(ionic liquid) and tetrahydroxydiboron as sources of B/N. The physicochemical properties of carbons and their chemical composition are much governed by the precursor in terms of its chemical nature and microstructure. Hence, the renewability and cost-effectiveness, along with the channel-like pores that are interconnected, are apparent advantages of using Balsa wood as a precursor for porous carbons of high conductivity.^15^ Additionally, owing to the PIL’s high boron and nitrogen contents, PILs act as an effective source of B and N, blending targeted heteroatoms into porous carbons. Furthermore, the PIL was reported to catalytically degrade biomass, inducing additional porous structures.^16^ Compared to other polymers, PILs exhibit superior thermal stability, ensuring a high carbonization yield.^17^ They are rich in diverse heteroatoms that contribute to carbon doping and can facilitate the uniform distribution of heteroatoms within the porous carbon matrix. The type of the cation and the anion of PILs is of key importance in creating small pores to accommodate catalytic active sites.^14^ Utilizing PIL-coated delignified wood slices as precursors allows the formation of a thin porous carbon membrane, effective for mass transport and thus catalytic activity. Importantly, serving as a macroscopic-sized heterogeneous catalyst, the carbonaceous membrane is readily recyclable by taking it out of the liquid mixture of the reaction.

In a representative synthetic procedure, a boron-containing compound tetrahydroxydiboron and the target PCMVImTFSI were mixed and dissolved in DMF. The used PCMVImTFSI has an apparent molar mass of 6.84 × 10^5^ g/mol, as determined by gel permeation chromatography. This mixture solution was coated onto the wood cell slice through wet-impregnation and it was then dried at 80 °C in an oven for 2 h to constant weight. Next, the composite membrane was placed in an aqueous NH_3_ solution (0.25 wt %) to develop pores in the PIL coating layer. It was wholly dried without any crack and carbonized under vacuum at 900 °C into the desirable carbon membrane product. PILs, renowned as surface active material, can effectively adhere to the wood surface through various intermolecular interactions, e.g., van der Waals forces and H bonding.^18^ Due to the ionic complexation between the tetrahydroxydiboron and the PILs, B can be integrated into the porous PILs’ layer homogeneously coating the porous wood surface.

**Figure 1:**
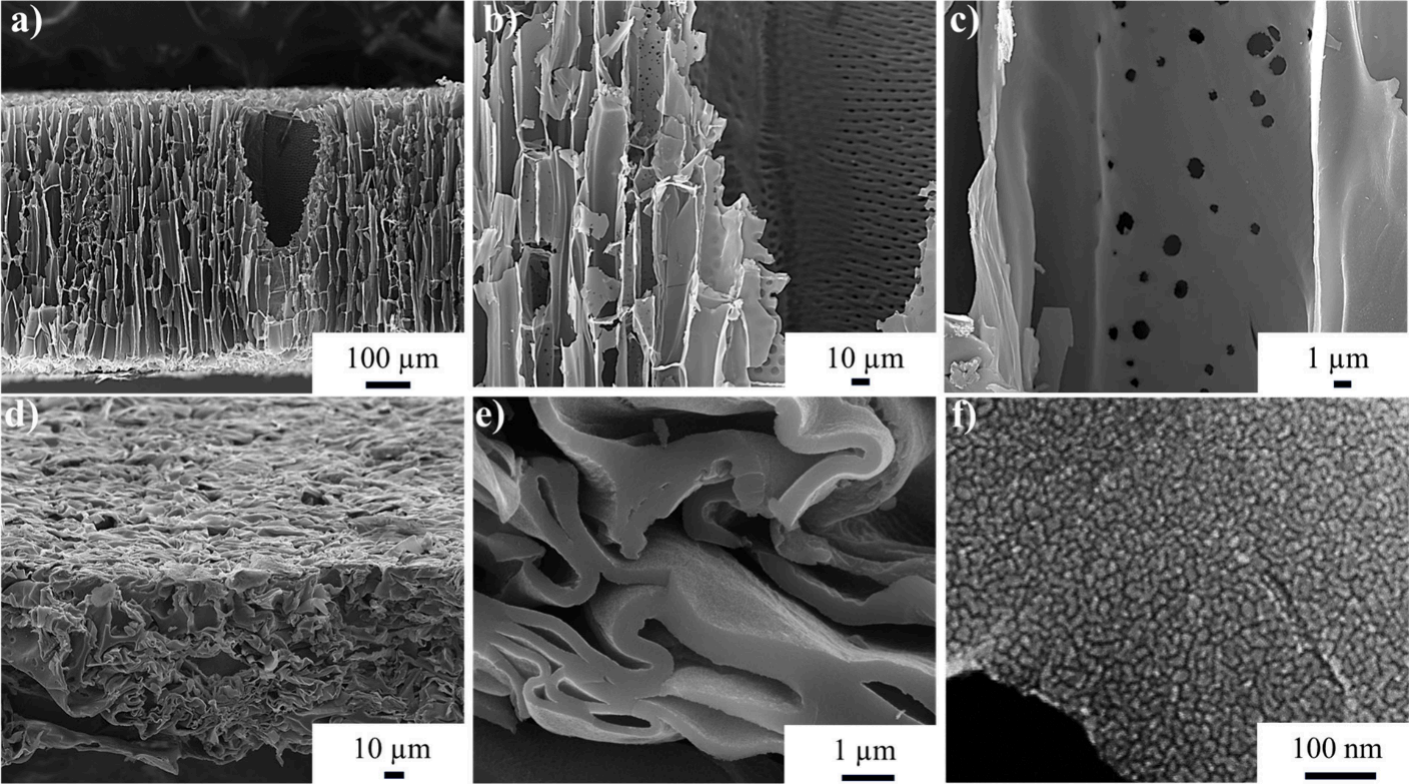
SEM images of the cross sections of (a–c) delignified wood and (d–f) the as-synthesized B/N–C at different magnifications.

Cross-sectional SEM images of delignified Balsa wood (Figure 1a, b, and c) illustrate the distinctive hierarchical microstructure, featuring extensive open channels such as xylem vessels and fibers-oriented perpendicular to the wood slice surface. They reveal that the cellular structures are oriented along the direction of growth of trees, and the xylem vessels present numerous micron-sized pores (pits) on the top of the inner surfaces, as depicted in Figure 1c. These channels, with diameters ranging from tens to hundreds of μm, play a crucial role in transporting nutrients, water, and ions from the bottom roots to above leaves,^19^ and are beneficial for the target porous carbon materials if successfully maintained along carbonization. To raise the electron conductivity of the carbon membrane, the PIL-coated delignified wood prior to carbonization was compressed, an action that densifies the cellular structure to increase the conductivity.^20^

The resulting carbon membrane obtained from Balsa wood subjected to mechanical pressing exhibits a distinct layered structure (Figure 1d and e). All cell walls are parallelly aligned well to each other and among closely positioned layers it generated tiny interstitial voids. Notably, numerous nanopores are observed on individual carbonized cell walls through SEM imaging (Figure 1f). This effect likely arises from a synergistic interplay between the selective removal of the lignin and hemicellulose components, and the subsequent conformal carbonization step.^21^ The open channels within the 3D porous carbon framework, along with nanopores in the carbon membrane wall, effectively reduce the diffusion length.^22^ This configuration of different pores leads to efficient and rapid mass transport to and from the active sites.^23^

**Figure 2:**
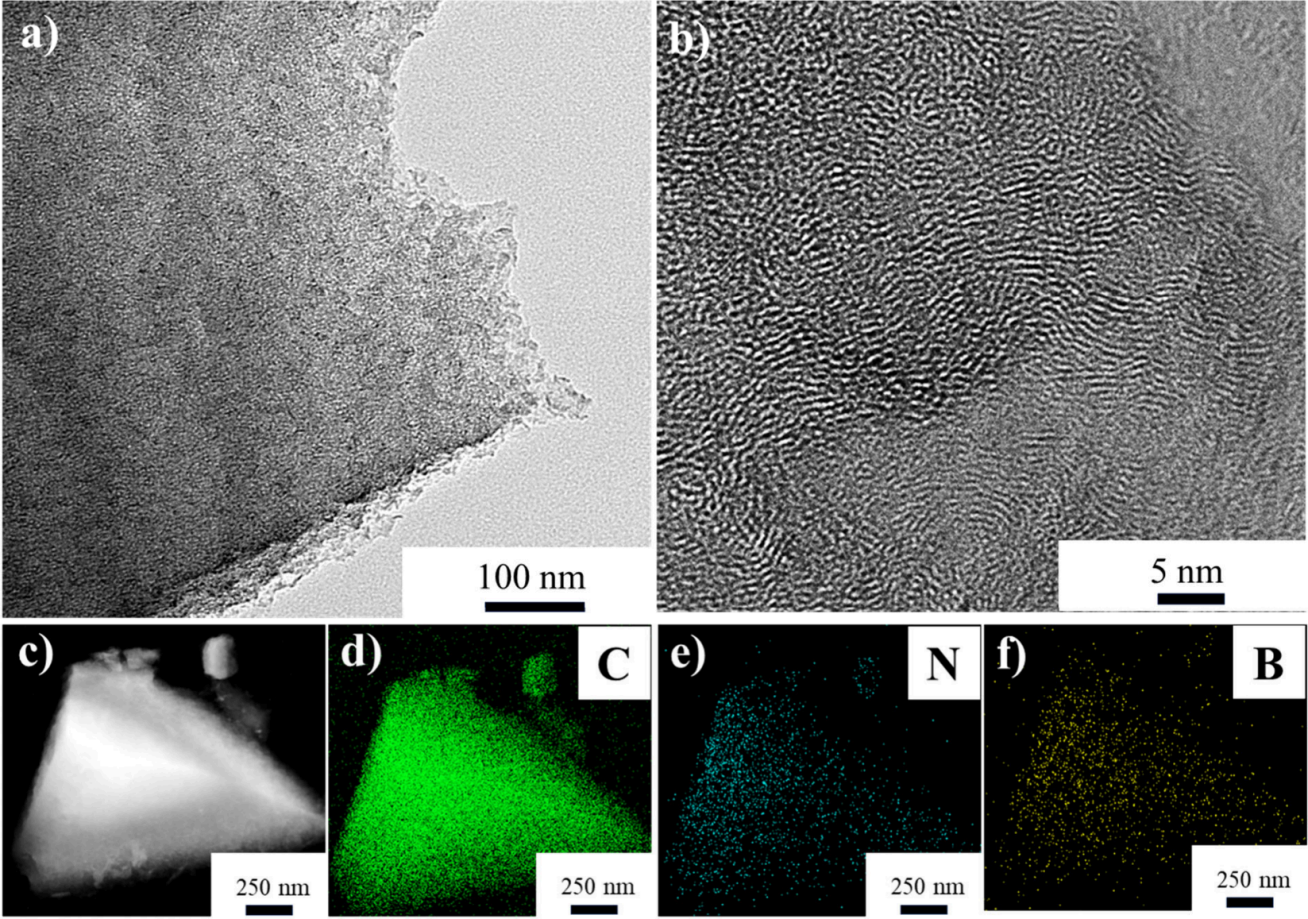
(a, b) High-resolution TEM images of B/N–C. (c–f) The related elemental mapping images by TEM of C, N, and B in the same sample.

Chemical treatment of wood is essential for the preparation of thin carbon membranes. Straightforward carbonization of the bulk wood without such treatment leads to fragile carbons that are unsuitable for shaping and forming cracks.^24^ Attempts to reduce the thickness of carbon membranes by cutting usually result in breakage due to mechanical stress. Delignification helps preserve the porous wood framework, enabling the creation of thin carbon membranes below 1 mm in thickness. Figure S2 shows a photograph of a crack-free thin carbon membrane of 96 ± 4 μm derived from PIL-coated wood. In a typical wood structure, cellulose, hemicellulose, and lignin build up cellulose fibril bundles that are encompassed by their intertwined matrix of both lignin and hemicellulose. The chemical treatment removes most of the lignin and hemicellulose components so that crystalline cellulose nanofibrils are better aligned *via van der Waals* forces and H bonding.^25^ Straightforward carbonization will decompose the amorphous lignin and hemicellulose components of natural Balsa wood, and generate cellulose nanofibrils in a random stacking mode without structural integrity.^26^ By contrast, carbonization of the delignified wood slice, which possesses a rearranged and connected cellulose framework, avoids the above-mentioned problem. The structural uniformity and integrity of the delignified wood during carbonization are well maintained, which minimizes mechanical stress inside the wood and thus replicates it is morphology well into the carbon product.^27^ To further study the morphology of the B/N–C, transmission electron microscopy (TEM) analysis has been conducted (Figure 2). The high-resolution TEM reveals that within the amorphous carbon matrix, the scattered nanoscale domains exhibit a discernible lattice spacing of approximately 0.36 nm, suggesting the existence of a graphitic phase. TEM analysis shows a uniform distribution of the B and N atoms throughout the carbon product. This observation aligns with the expectations of using the molecular dopant to introduce heteroatoms (Figure 2c, d, e, and f).

The chemical structure of PILs as precursors appears as the principal factor controlling the carbonization yield. As PILs have high thermal stability due to the IL species, the PIL-coated delignified wood in our study shows a high yield around 21%. Furthermore, the introduction of heteroatoms, especially N, can change not only the bulk but also the surface properties of carbon materials and thus enhance their adaptability for applications in catalysis, sorption, and so on. In this investigation, the imidazolium units of PILs function as the sole source of N. Elemental analysis (EA) reveals a nitrogen content of around 5.8 ± 0.1 wt % for B/N–C sample. Acknowledging that carbon’s electronic structure is sensitive to the doping pattern, there is a rising interest in creating functional carbons through the design of heteroatom dopants via doping with more than one type of heteroatoms. As an example, the B/N dopants in graphitic carbons could move the Fermi level toward the valence band, enhancing pore interface wettability. Such alteration can improve both charge storage and transfer inside the carbon matrix.^28^ In this regard, apart from nitrogen, the amount of boron should be analyzed carefully. The measured content of boron in B/N–C, is 0.28 ± 0.04 wt %, which was measured via inductively coupled plasma optical emission spectroscopy (ICP-OES).

**Figure 3:**
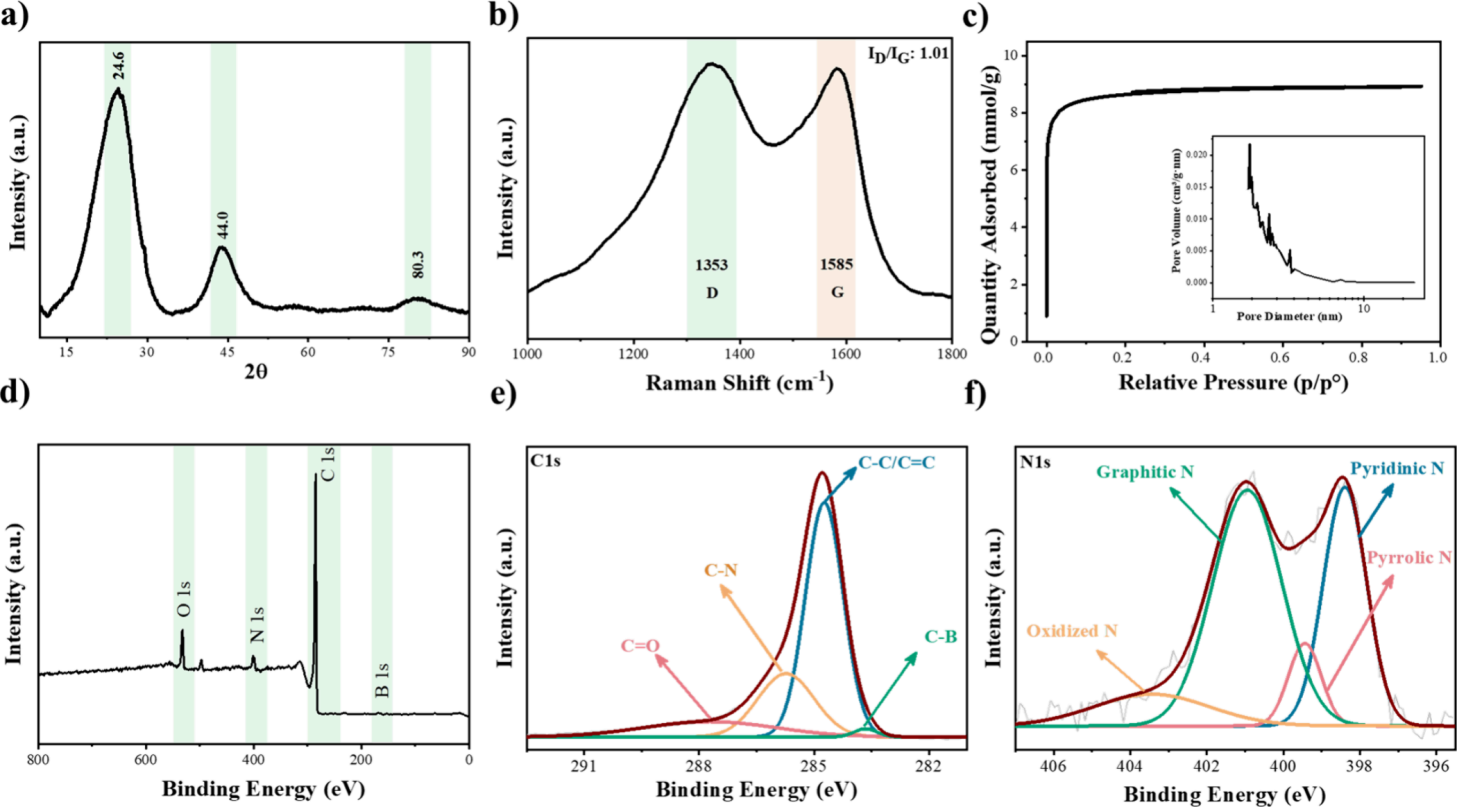
Analysis of the structure of the obtained B/N–C sample. (a) XRD diagram; (b) Raman spectrum; (c) nitrogen adsorption/desorption isotherms measured at 77 K; (d) XPS survey full spectrum; (e, f) corresponding high-resolution C 1s and N 1s spectra, respectively.

X-ray diffraction (XRD) analysis has been conducted to evaluate the content and phase structure of the B/N–C sample (Figure 3a). As illustrated in Figure 3a, three notable peaks appear at 24.6°, 44.0°, and 80.3°. The possible long-range order in the carbon products is implied by the appearance of the peak at 80.3°. Minor changes in peak height, breadth, and shift suggest the presence of defects in the structure.^29^ In this case, a discernible graphitic peak (002) associated with *sp*^2^ hybridized carbon consistently appeared at 2θ ∼ 24.6°, standing for an interlayer spacing of 0.36 nm. The interlayer spacing beyond 0.34 nm for fully graphic carbons signals the existence of substantial defects within the graphitic phase, disrupting the perfect stacking of graphitic sheets.^30^ Incorporating heteroatoms into carbon structure, whether in the case of single-heteroatom doping or codoping, can profoundly affect the graphitic structure. When heteroatoms are bonded to carbon atoms covalently and homogeneously integrated into the carbon matrix, it introduces disruptions to the *sp*^2^ carbon. It results in the generation of defects, deformation of the graphitic planes, and enlargement of the interlayer spacing. To note, structural defects play an important role in disrupting the symmetry of charge density or spin density in carbon materials, leading to the localization of electrons and the creation of active sites.^31^ Furthermore, the expanded interlayer spacing improves the intercalation of guest species into the graphitic phase. These structural modifications collectively create a favorable environment for peroxidase-like catalytic activity.

Raman spectroscopy serves as an impactful instrumental method for investigating the phase structure information on carbon materials. Information regarding the level of graphitization degree, the presence of structural defects, and the dopants of the carbon can be determined from the intensity and position of the Raman peaks.^32^ In Figure 3b, two discernible bands are observed at 1353 and 1585 cm^–1^. They are designated, respectively, as the D-band and the G-band. The former is associated with a disordered arrangement of carbon atoms and structural defects; the latter is related to ordered graphitic structures.^33^ The I_D_/I_G_ ratio, representing the level of structural defects and disorder degree of the carbon sample, is 1.01. In general, carbon materials that undergo heteroatom doping show a higher I_D_/I_G_ value than undoped ones (in contrast to graphite, where the I_D_/I_G_ is typically less than 0.1^34^). This elevation in the degree of disorder is attributed to the incorporation of heteroatoms, leading to notable variation in bond length and angle, as well as the electronic structure of the carbon sample. To note, the I_D_/I_G_ ratio close to 1 demonstrates the sample’s effective graphitization. Importantly, the enhancement of graphitization assists the electron flow within the carbonized porous carbons.^35^ In our specific sample, the apparent conductivity is measured as high as 4700 ± 50 S/m, despite the presence of rich pores. Consequently, this carbon material has the potential to function as a conductive sample with favorable peroxidase-like catalytic activity.

In general, the catalytic activity is significantly influenced by porous structure and contact area.^36^ Under equivalent conditions, superior catalytic performance is observed in materials possessing a larger surface area. A larger surface area can hold a bigger number of active sites. As a result, the catalyst can interact more effectively with the substrate and successfully oxidize the TMB, given that the active sites are accessible. The analysis of surface area and pore size distribution has been carried out using the nitrogen sorption isotherm at 77 K (Figure 3c). The isotherm exhibits a shape consistent with IUPAC-type I isotherm. The specific surface area determined by the Brunauer–Emmett–Teller (*S*_BET_) equation and the pore volume of B/N–C have been confirmed to be 618 m^2^g^–1^ and 0.43 cm^3^g^–1^, respectively. In addition, the pore size distribution plot in the inset validates the dominance of micropores below 2 nm. The existence of plentiful micropores obviously is essential to host dense active sites for catalysis, as enabled by a high surface-to-volume ratio.^37,38^ Nonetheless, the complete catalytic power of these micropores is commonly impeded by pronounced resistance in diffusion via pores under 2 nm. Successfully addressing this challenge is demonstrated by the inclusion of macropores and channels, as evidenced by the SEM image in Figure 1d-f. A hierarchical arrangement of pores proves advantageous in catalysis by establishing connections between micropores. This arrangement ensures a high catalytic activity and simultaneously a sufficient mass flow. It is noteworthy that the thermal degradation of the TFSI^–^ anion typically in an ion cluster form in the PILs/wood composite serves as a main factor for creating micropores in these porous carbons.^39^ In the carbonization procedure, a vacuum condition facilitates the effective elimination of TFSI^–^, and generates micropores. Nevertheless, it is crucial to highlight that an applied vacuum that is too high, may improve graphitization of the B/N–C which meanwhile reduces the micropore formation.^40^

We have performed a further examination of the surface of B/N–C catalyst using XPS to study its constituent elements and the corresponding electronic states. The existence of C, N, O, and B elements was verified by the survey spectrum of B/N–C (Figure 3d). Furthermore, the survey spectrum reveals surface concentrations of 5.48 atom % for N and 1.1 atom % for B. It is important to acknowledge that the quantified elemental content derived from XPS analysis appears to be different from their bulk. This discrepancy is ascribed to the inherent limitation of XPS in accessing atomic sites buried in the carbon matrix, owing to the restricted penetration depth of X-ray in XPS, typically up to ∼10 nm.^41^ For an insight view, the high-resolution C 1s spectrum can be readily deconvoluted into four distinct peaks (Figure 3e). The primary peak at 284.7 eV, of the highest intensity (in an abundance of 61 atom %) is assigned to the C–C/C=C bond, proving the presence of graphitic carbon. The prominent peak at 285.7 eV is indicative of the nitrogen-binding carbon (C–N). The one located at 287.7 eV stands for the C–O bond, and it is likely due to contamination of the sample surface. Lastly, the C 1s peak located at 283.7 eV corresponds to C–B bonds.^7^Figure 3f illustrates the N 1s survey spectrum. The signal is readily deconvoluted to four at 398.4, 399.4, 400.9, and 403.5 eV, which can be assigned to the pyridinic, pyrrolic, graphitic, and oxidized N. This analysis elaborates on the bonding nature of N with carbon atoms. Diverse nitrogen configurations within a carbon framework demonstrate distinct functionalities. For instance, the graphitic N site (in a *sp*^2^ hybridization), constituting approximately 49.5 atom % in our case, contributes its lone electron pair into the conjugated π-system, resulting in a partial positive charge. On the contrary, pyridinic N (31.3 atom %), characterized by *sp*^2^ hybridization, actively directs one electron in the *p*-orbital to the aromatic π system, exhibiting a pronounced electron-donating nature.^42^ The simultaneous presence of graphitic and pyridinic N possesses the capacity to enhance electron circulation because of their slightly smaller atomic size and larger electronegativity than carbon. Consequently, the amalgamation of graphitic carbon bearing a considerable amount of graphitic and pyridinic N in the carbon matrix can synergistically assist in cleaving the O–O bond in H_2_O_2_.^43^

**Figure 4:**
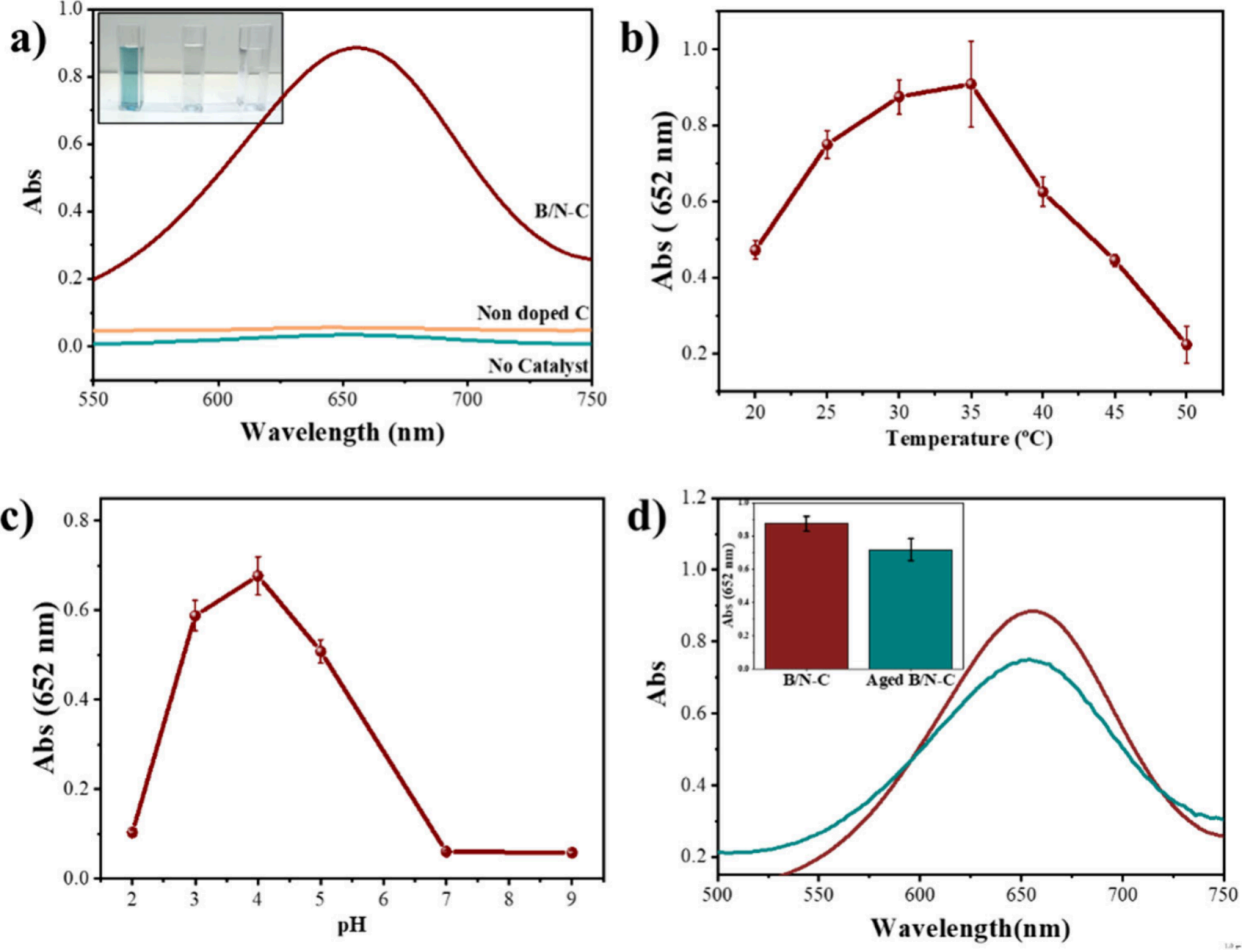
(a) UV–Vis absorbance spectroscopic analysis of three reaction systems (oxidation of TMB by H_2_O_2_, catalyzed by B/N–C) in the wavelength range of 550–750 nm in an acetate buffer solutions at pH = 4.0. (b, c) Plot of absorption intensity (recorded at 652 nm) against temperature and pH value, respectively, of the B/N–C catalyst, showing the temperature-/pH-dependence of the peroxidase-like activity of B/N–C. (d) UV–Vis absorbance spectra of two reaction systems (oxidation of TMB by H_2_O_2_) in the wavelength range of 500–750 nm in an acetate buffer solution at pH = 4.0, catalyzed by freshly prepared B/N–C catalyst and by the aged B/N–C catalyst after six-month storage at ambient temperature at 652 nm.

In investigating the influence of the codoping effect of heteroatoms on the chemical properties, the B/N–C sample due to its peroxidase-like activities was explored as an artificial enzyme by using the TMB as a substrate in its reaction with H_2_O_2_. In a standard procedure, the original colorless liquid reaction mixture undergoes a transformation to a blue color, marked by a distinctive UV–vis absorbance peak at 652 nm. This peak emerges due to the presence of oxidized TMB (referred to as ox-TMB), similar to the characteristic observation in the well-established horseradish peroxidase (HRP) reaction.^44^ The aqueous solution of TMB with only H_2_O_2_ demonstrated no noticeable UV–vis absorbance at 652 nm, remaining in an unaltered state (Figure 4a), indicating the absence of any observable oxidation reaction. Nevertheless, adding B/N–C to the reaction of H_2_O_2_ and TMB, a blue coloration was recognized (Figure 4a). It supports the capacity of our artificial enzyme to effectively decompose H_2_O_2_, which is responsible for starting the oxidative reaction of TMB into ox-TMB, a process that can be monitored by the absorption peak at 652 nm in its UV–vis spectra.^45^ Furthermore, a control test was conducted to assess the activity of the carbon without heteroatom, produced from the carbonization of the delignified wood only. The outcome affirms that the presence of heteroatom doping is indispensable in this application. As a result, the incorporation of multiple heteroatoms exhibits the capability to magnificently improve the catalytic activity of B/N–C over a wider range compared to nondoped carbonous materials and even nitrogen-doped carbon materials (Figure S3).^46^ This advancement is ascribed to a synergistic effect, and more catalytic active sites and defects in the carbon material, due to the introduction and interplay of multiple heteroatoms. Co-doping carbon by B and N, where N (χ_p_ = 3.04) exhibits a higher electronegativity and B (χ_p_ = 2.04) with lower electronegativity than C (χ_p_ = 2.55), results in a distinctive electronic structure marked by a coupling effect among heteroatoms. Such an effect was documented to markedly elevate the catalytic activity of carbonous catalysts codoped with dual heteroatoms in comparison to their nondoped carbon.^47^ The theoretical investigation has revealed that the codoping arrangement in the atomic form of N–C–B incorporates the electron-withdrawing characteristics of N, encouraging polarization in the neighboring carbon atom and enabling extra electron donation to the nearby boron atom. Such a phenomenon results in higher electron occupancy and developed overall catalytic activity.^48^

To examine the reaction’s dependency on TMB content, the optimized concentration was calculated and maintained throughout all procedures (Figure S4). One critical variable influencing catalytic reactions is temperature. Therefore, we have explored how the catalytic activity of B/N–C is affected by the reaction temperature in the range of 20–50 °C. As depicted in Figures 3b and S5, our peroxidase catalyst exhibits optimal performance at 35 °C, a temperature that closely mirrors that of the human body. This feature improves its suitability for detection in biological samples. It is noteworthy that the decline in activity noticed beyond 35 °C aligns with phenomena observed previously.^49^ We studied the pH impact on catalytic activity within the pH range from 2.0 to 9.0 (Figures 4c and S6). Under highly acidic conditions (pH 2.0), a light blue color was detected. At either pH 3.0 or pH 5.0, a mild blue solution color, representing ca. 60% of the activity in the reaction system, was obvious. Remarkably, at pH 4.0, a blue solution color was noticeable, implying an optimal performance for the catalyst at this pH level. This observed behavior aligns with previous studies, where HRP demonstrated analogous characteristics.^50^ To evaluate the robustness of the B/N–C in catalytic operation, we aged the samples at room temperature for a duration of 6 months, and subsequently, their UV–vis absorbance spectra were measured. The results revealed a marginal decrease in absorbance, and the carbonaceous catalyst maintained a resilient performance throughout the storage period (Figure 4d). The recycling result for the catalyst (Figure S7) showed its beneficial performance after 3 continuous uses.

**Figure 5:**
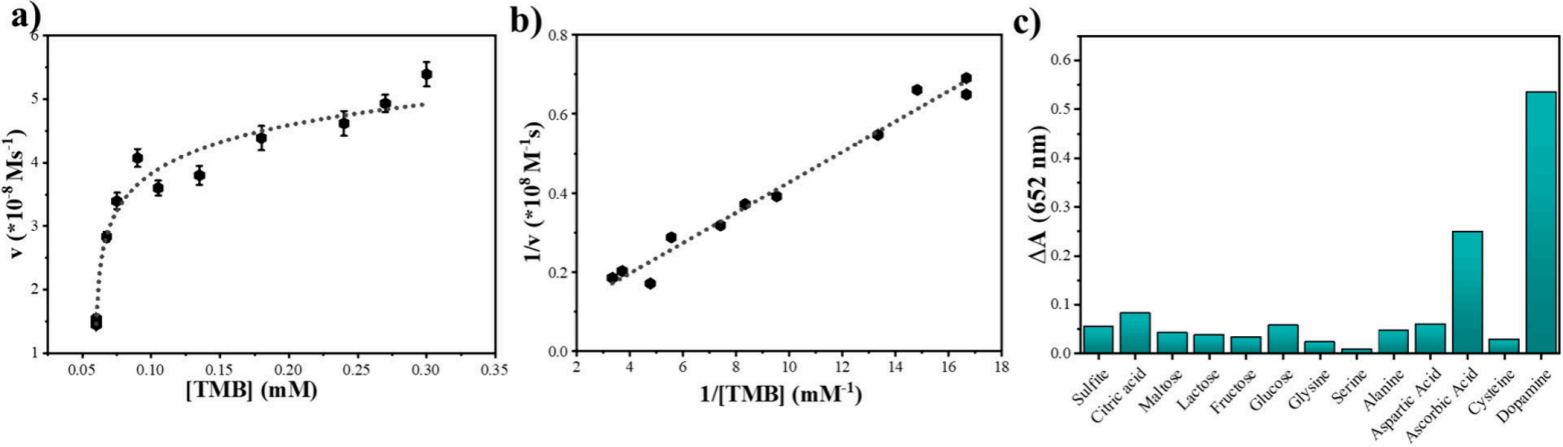
Study of steady-state kinetics of B/N–C catalyst for the catalytic oxidation of TMB by H_2_O_2_. (a) Michaelis–Menten curve for TMB substrate. (The H_2_O_2_ concentration was fixed, and the TMB concentration was changed.) (b) Lineweaver–Burk plot for TMB substrate. (c) The Δ*A* values obtained in the B/N–C–TMB–H_2_O_2_ catalytic reactions at 652 nm for different interferential compounds.

To determine the steady-state kinetic properties of the mimetic peroxidase reaction using B/N–C as the catalyst, the TMB concentration in the reaction was varied and the H_2_O_2_ content was unchanged. Its catalytic performance was extensively examined via kinetic analysis, using the concentrations of TMB as variables. Figure 5a exhibits a typical Michaelis–Menten curve, from which the corresponding Lineweaver–Burk plot was derived (Figure 5b). The initial reaction velocity of ox-TMB can be readily determined from the UV–vis absorbance data, and by using the Beer–Lambert law (Equation 2):
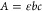
2In Equation 2, ***A*** stands for the absorbance, ***ε*** for the molar absorptivity coefficient (here 39,000 M^–1^cm^–1^ was taken for TMB at λ = 652 nm), ***c*** for substrate concentration, and ***b*** for the length of light path.

We derive the Michaelis constant (K_m_) and maximum rate achieved by the catalytic system (*V*_max_) from the following Equation 3:
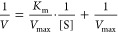
3In this investigation, ***V*** and ***[S]*** represent the reaction velocity, and the substrate concentration, respectively. It is well-established that the catalytic performance of a catalyst hinges on its values of ***K***_***m***_ and ***V***_***max***_. Typically, a lower ***K***_***m***_ speaks for a higher affinity of the catalyst to a substrate, while a larger ***V***_***max***_ value denotes a better efficiency in TMB oxidation by H_2_O_2_. For our catalyst, the ***K***_***m***_ and ***V***_***max***_ values calculated for TMB are 0.87 mM and 22.9 × 10^–8^ Ms^1–^, respectively. It is evident that B/N–C presents a much larger ***V***_***max***_ for TMB than previously reported artificial enzymes (as detailed in Table S1). This substantial difference highlights the superior efficiency of B/N–C in TMB oxidation and its advanced peroxidase-like catalytic activity. Our B/N–C catalyst provides undoubtedly large ***V***_***max***_ values, further emphasizing its superior catalytic efficiency. This enhancement could be attributed to a large surface area, dense hierarchical pores, and dual heteroatoms, which provide sufficient active sites for catalyzing TMB oxidation and result in a large ***V***_***max***_ value. Here, the ***K***_***m***_ value is comparable to that of HRP (0.41 mM) and indicates a good affinity of our catalyst to TMB. Furthermore, the rapid colorimetric response of the B/N–C system, with an obvious color difference observed in less than 5 min, underscores its productivity and suitability for rapid visual colorimetric tests, a critical factor in practical applications. In addition, the steady-state kinetic experiments have been tested for H_2_O_2_ as the substrate, and ***K***_***m***_ and ***V***_***max***_ were measured, 57.97 mM and 9.7 × 10^–8^ Ms^1–^ respectively (Figures S8 and S9).

Additionally, we found that dopamine slows down the peroxidase-like activity of our catalyst selectively and effectively. This discovery can be utilized for the development of a dopamine label-free colorimetric assay (Figure 5c). Although the primary mixture containing B/N–C exhibited substantial and immediate catalytic activity, its peroxidase-like function was significantly suppressed upon exposure to and interaction with the dopamine molecule. The drop in peroxidase-like activity is ascribed to a competition of dopamine of adsorption onto the catalyst, leading to alterations of the catalyst surface. Furthermore, the colorimetric technique shown here demonstrated outstanding sensitivity in detection specifically of dopamine in the presence of other interfering substances.

## 4 Conclusion

In this study, a conformal carbonization methodology was applied to produce hierarchically porous B/N codoped carbonaceous catalysts, exhibiting exceptional peroxidase-like activity. These catalysts were derived from delignified wood slices as carbon source, which were coated with a heteroatom-rich poly(ionic liquid) to facilitate adjustment of heteroatom dopants in the resulting carbon membranes. The introduction of B alongside N, coupled with the hierarchical porous structure, led to the creation of more accessible defects and active sites. The resulting B/N–C catalyst, characterized by its distinctive interconnected and oriented porous structure, and uniform heteroatom codoping, demonstrated remarkable intrinsic peroxidase-like catalytic activity with notable stability. In comparison to prior studies, B/N–C demonstrates improved catalytic behavior and elevated ***V***_***max***_ values, speaking for high peroxidase-like activity and enhanced substrate affinity.

In short, our study presents a facile and efficient approach for fabricating metal-free carbonaceous doped with heteroatoms. This methodology is applicable to synthesizing diverse functional carbonaceous materials, including but not limited to artificial enzymes. The confirmed potential of these materials is expected to extend to applications in healthcare and environmental rehabilitation.

## Supporting Information Available

Proton nuclear magnetic resonance spectrum of the PIL, photographs of the carbon membrane, analytic data for the peroxidase-like activity, and comparison of the B/N–C catalyst with studies in literature (PDF)
