# Supplementary material for: Poly(ionic liquid)/Wood Composite-Derived B/N-Codoped Porous Carbons Possessing Peroxidase-like Catalytic Activity
Source: ACS Omega. 2024 Sep 3;9(37):39170–9. doi: 10.1021/acsomega.4c06102 (PMC11411521; doi:10.1021/acsomega.4c06102)
Supplement: Supplementary file 1 — ao4c06102_si_001.pdf [file ao4c06102_si_001.pdf]

Supplementary Information

Poly(ionic liquid)/wood composite-derived B/N-codoped porous carbons possessing peroxidase-like catalytic activity

*Sadaf Saeedi Garakani, Kanglei Pang, Elnaz Tahavori, Anuja Pradip Nawadkar, Özlem Uguz Neli, and Jiayin Yuan**

Department of Materials and Environmental Chemistry, Stockholm University, Stockholm 10691, Sweden

*Corresponding author

E-mail: jiayin.yuan@mmk.su.se

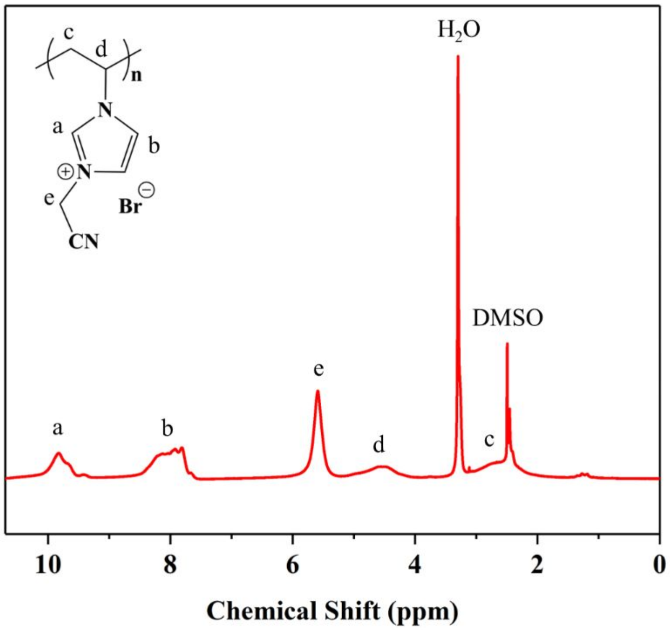

Figure S1 ^{1}H-NMR spectrum and its peak assignment of the PIL poly(1-cyanomethyl-3-vinylimidazoluim bromide) (PCMVImBr), which was used as additive for the porous polymer membrane fabrication. NMR solvent: DMSO-*d*$_6$.

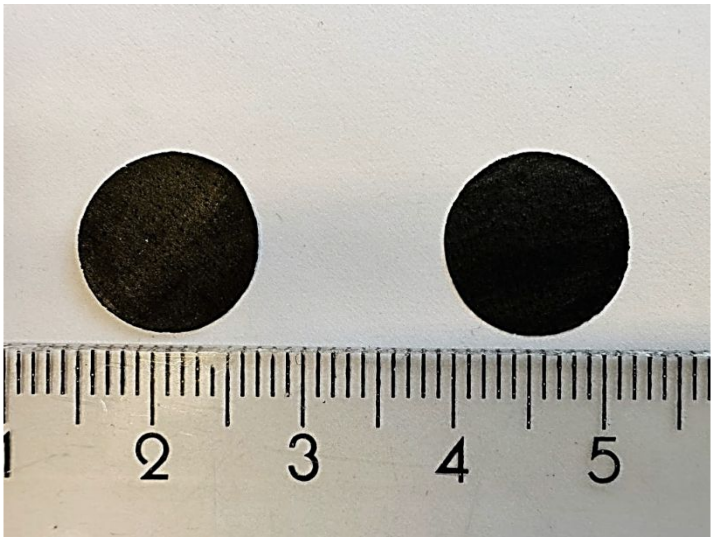

Figure S2 Photograph of two B/N-C crack free membranes.

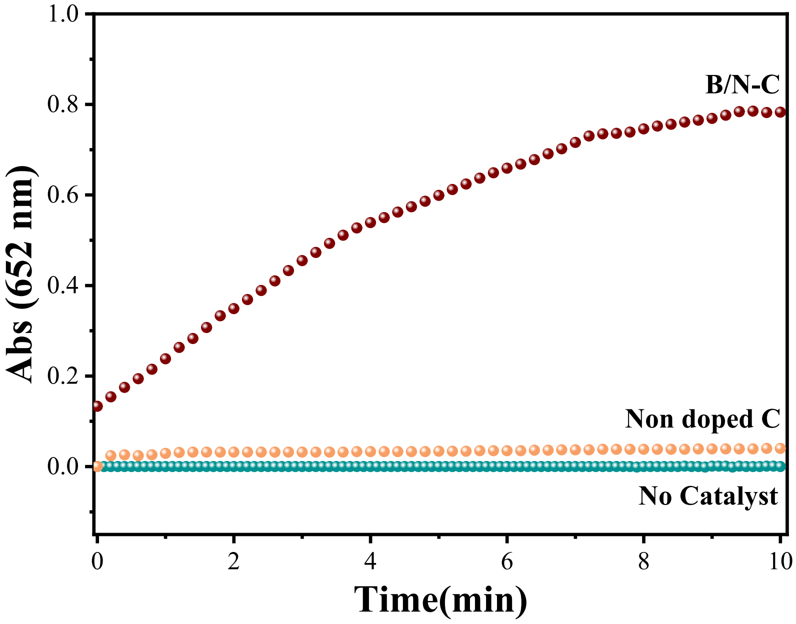

Figure S3 Absorbance at 652 nm of the ox-TMB recorded versus time at room temperature in acetate buffer solutions at pH = 4.0.

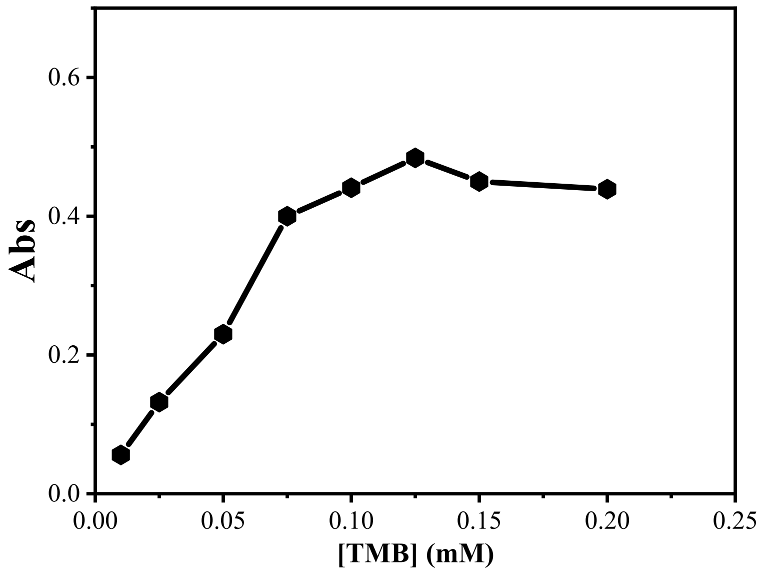

Figure S4 Absorbance at 652 nm of the ox-TMB recorded versus TMB content at room temperature in acetate buffer solutions at pH = 4.0.

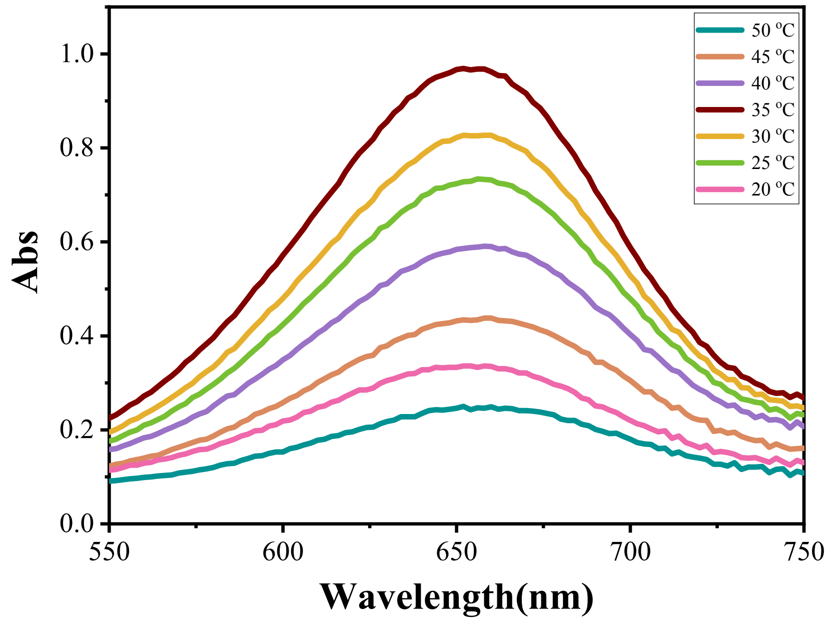

Figure S5 UV−Vis absorbance spectra (only the 550-750 nm range was presented here) of the ox-TMB recorded at various temperatures in acetate buffer solutions at pH = 4.0.

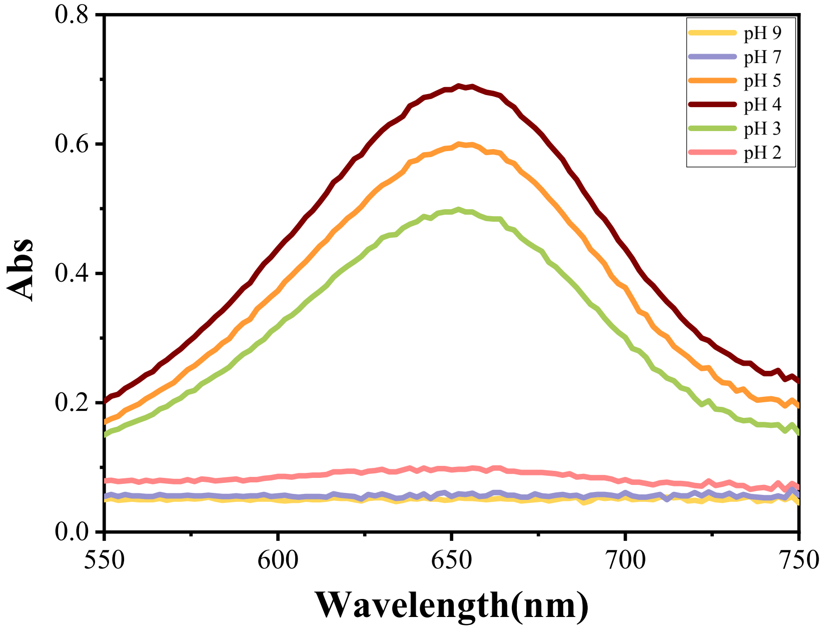

Figure S6 UV−Vis absorbance spectra (only the 550-750 nm range was presented here) of the ox-TMB recorded at various pH at room temperature.

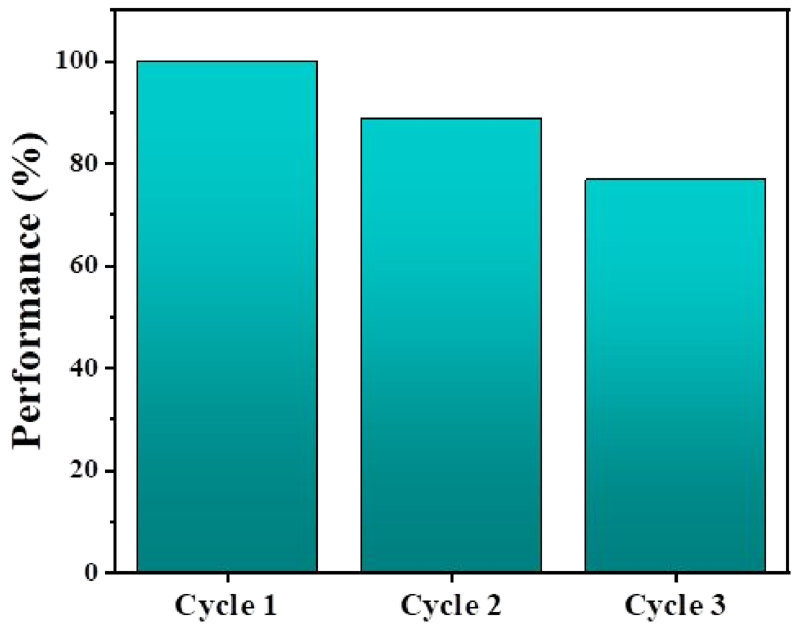

Figure S7 The stability test of the catalyst after 3 cycles of use.

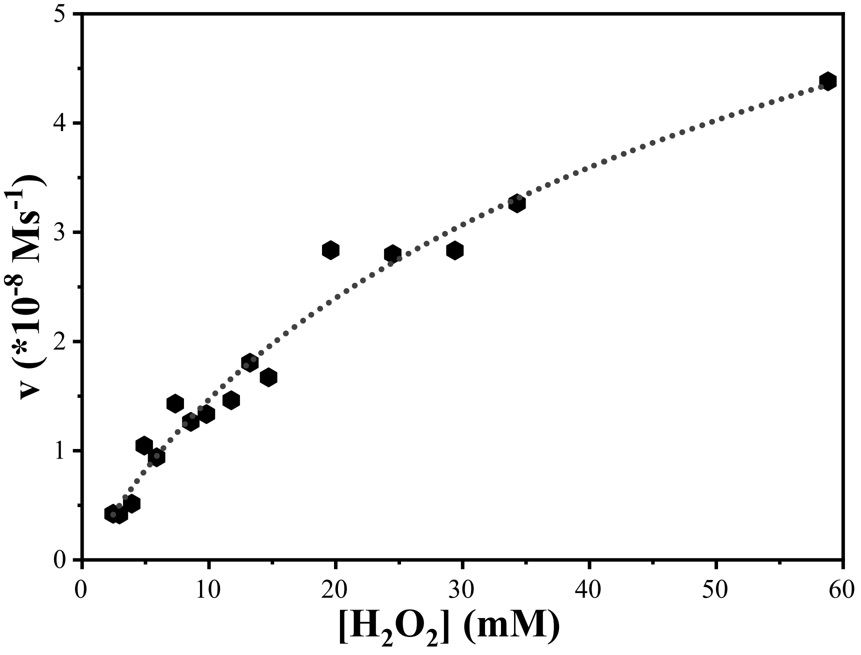

Figure S8 Steady-state kinetic experiments of B/N–C for catalytic tests. Above is the Michaelis–Menten curve for H_2O_2 substrate.

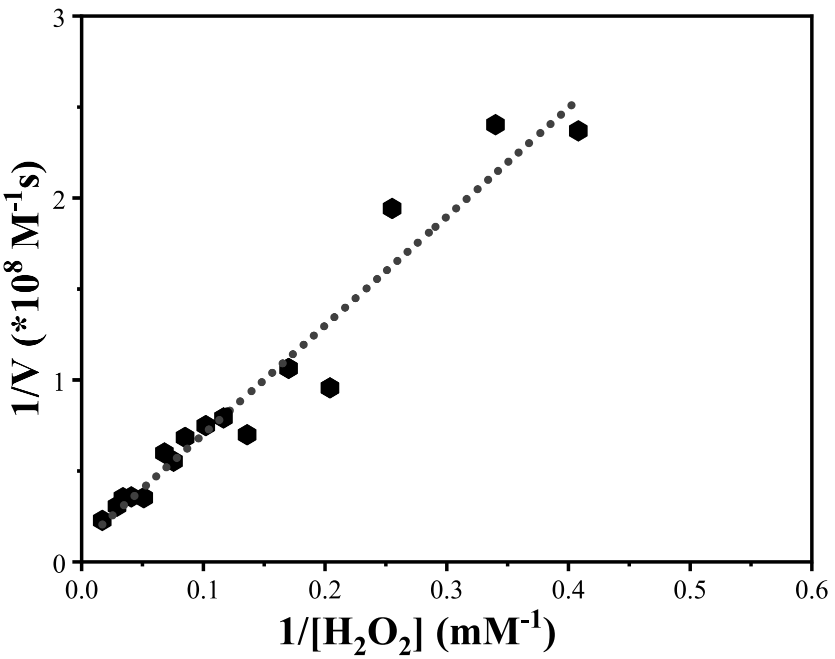

Figure S9 Lineweaver–Burk plot for H_2O_2 substrate.

Table S1 Comparison of the apparent Michaelis constant (K_m) and maximum reaction rate (V_{max}) between our work and data of other groups reported in literature.

**Catalyst**	**Substrate**	**K_m (mM)**	**V_{max} (10^{-8} M s^{-1})**	**Reference**
HRP	TMB	0.43	8.71	[2]
DHPC@CS-AgNPs	TMB	0.0165	4.6512	[3]
Fe_3O_4	TMB	0.098	3.44	[4]
B/N-C	TMB	0.097	6.5	[5]
Au/Co_3O_4-CeO_x NCs	TMB	0.1219	0.8577	[6]
WSe_2 nanosheets	TMB	0.433	1.43	[7]
WS_2/rGO	TMB	22.406	0.9606	[2]
AKCN	TMB	0.60	6.78	[8]
Fe_3C/NGr	TMB	0.25	8.26	[1]
**B/N-C**	**TMB**	**0.87**	**22.9**	**This Work**

References

1 S. Wu, H. Huang, X. Feng, C. Du and W. Song, Facile visual colorimetric sensor based on iron carbide nanoparticles encapsulated in porous nitrogen-rich graphene, *Talanta*, 2017, **167**, 385–391, , DOI:10.1016/j.talanta.2017.02.003.

2 S. Keerthana, A. Rajapriya, C. Viswanathan and N. Ponpandian, Enzyme like-colorimetric sensing of H2O2 based on intrinsic peroxidase mimic activity of WS2 nanosheets anchored reduced graphene oxide, *J. Alloys Compd.*, 2022, **889**, 161669, , DOI:10.1016/j.jallcom.2021.161669.

3 S. Zhang, S. Wei, H. Cheng and B. Rong, A highly sensitive colorimetric sensor for Hg2+ detection based on the oxidative enzyme mimics-like activity of hierarchical porous carbon@chitosan-modified silver nanoparticles, *J. King Saud Univ. - Sci.*, 2020, **32**, 1265–1271, , DOI:10.1016/j.jksus.2019.10.015.

4 L. Gao, J. Zhuang, L. Nie, J. Zhang, Y. Zhang, N. Gu, T. Wang, J. Feng, D. Yang, S. Perrett and X. Yan, Intrinsic peroxidase-like activity of ferromagnetic nanoparticles, *Nat. Nanotechnol.*, 2007, **2**, 577–583, , DOI:10.1038/nnano.2007.260.

5 S. S. Garakani, A. Sikdar, K. Pang and J. Yuan, Poly (ionic liquid) -derived metal-free heteroatom co-doped porous carbons with peroxidase-like activity, *Appl. Mater. Today*, 2024, 102081, , DOI:10.1016/j.apmt.2024.102081.

6 H. Liu, Y. Ding, B. Yang, Z. Liu, Q. Liu and X. Zhang, Colorimetric and ultrasensitive detection of H2O2 based on Au/Co3O4-CeOx nanocomposites with enhanced peroxidase-like performance, *Sensors Actuators, B Chem.*, 2018, **271**, 336–345, , DOI:10.1016/j.snb.2018.05.108.

7 T. M. Chen, X. J. Wu, J. X. Wang and G. W. Yang, WSe2 few layers with enzyme mimic activity for high-sensitive and high-selective visual detection of glucose, *Nanoscale*, 2017, **9**, 11806–11813, , DOI:10.1039/c7nr03179c.

8 P. Zhang, D. Sun, A. Cho, S. Weon, S. Lee, J. Lee, J. W. Han, D. P. Kim and W. Choi, Modified carbon nitride nanozyme as bifunctional glucose oxidase-peroxidase for metal-free bioinspired cascade photocatalysis, *Nat. Commun.*, 2019, **10**, 1–14, , DOI:10.1038/s41467-019-08731-y.
